# Cardiovascular Implications of the Enhanced Games: Performance Enhancing Drugs in Competition and Recreation

**DOI:** 10.1186/s40798-026-01026-9

**Published:** 2026-04-24

**Authors:** Marco Vecchiato, Stefano Palermi, Mark Zamodics, Mate Babity, Mani Eftekhari, Rohith Ryali, Justin Luk, Atta Taseh, Soheil Ashkani-Esfahani, Gergo Merkely, Vencel Juhasz

**Affiliations:** 1 Sports and Exercise Medicine Division, Department of Medicine, University of Padova, Via Giustiniani 2, Padova, 35128 Italy; 2 Department of Theoretical and Applied Sciences, eCampus University, Novedrate, 22060 Italy; 3 Sports Medicine and Cardiovascular Rehabilitation Unit, AULSS3 Noale Hospital, Venezia, 30033 Italy; 4 Unicamillus Saint Camillus International University of Health Sciences, Via di Sant’Alessandro 8, Rome, 00131 Italy; 5 Heart and Vascular Center, Semmelweis University, Varosmajor st. 68, Budapest, 1122 Hungary; 6 Foot & Ankle Research and Innovation Lab (FARIL), Department of Orthopaedic Surgery, Mass General Brigham, Harvard Medical School, 158 Boston Post Rd, Weston, MA 02493 USA; 7 Foot and Ankle Division, Department of Orthopaedic Surgery, Mass General Brigham, Harvard Medical School, 55 Fruit St, Boston, MA 02114 USA; 8 Department of Orthopaedic Surgery, Mass General Brigham, Harvard Combined Orthopaedic Residency Program (HCORP), Harvard Medical School, 55 Fruit St, Boston, MA 02114 USA; 9 Department of Orthopedic Surgery, Semmelweis University, Ulloi rd. 78/b, Budapest, 1082 Hungary; 10 Cardiovascular Imaging Research Center, Massachusetts General Hospital & Harvard Medical School, Cambridge St 165, Ste 400, Boston, MA 02114 USA

**Keywords:** Performance enhancement, The enhanced games, Cardiovascular risk, Athlete’s heart, Sudden cardiac death, Cardiac remodeling

## Abstract

**Background:**

The Enhanced Games (TEG) initiative—an event that permits the off-label use of FDA-approved drugs for performance enhancement under medical supervision—represents a revolutionary yet highly controversial disruption in modern sport. Although the excessive use of certain performance-enhancing drug (PED) classes is associated with clear health risks, current evidence on PED-related cardiovascular (CV) risk is primarily derived from retrospective reports, small cohorts, or illicit use, leaving major gaps in mechanistic understanding and dose–response relationships in performance enhancement, well-being, and rehabilitation purposes.

**Main:**

This review synthesizes existing data on the ergogenic mechanisms and CV toxicity of key PED classes relevant to TEG athletes, including anabolic–androgenic steroids (AAS), growth hormone and IGF-1, erythropoiesis-stimulating agents (ESAs), stimulants, β₂-agonists, diuretics, metabolic modulators, and emerging incretin-based weight management therapies. Across these agents, ergogenic effects are inconsistently demonstrated, whereas CV harm is not rare, and may be cumulative and irreversible. AAS and ESAs exhibit the strongest ergogenic signals but are also associated with myocardial remodeling, arrhythmia, and thrombotic events. Other agents provide limited or unclear performance benefits yet may disrupt autonomic balance, metabolism, or myocardial integrity. With the emergence of availability through compounding pharmacies and a rapid increase in PED use among the general population, there is an urgent need for high-quality, prospective data to inform about health risks. Since the recreational and well-being use of PEDs is on the rise among the general population, PEDs’ dose-dependent detrimental effects must be carefully evaluated. By applying rigorous pre-participation screening and long-term follow-up, TEG may provide high-quality, longitudinal data not previously available with PEDs.

**Conclusion:**

TEG’s potential to provide valuable scientific insights should not be interpreted as a proof-of-concept for safe, extreme-level performance enhancement, but rather as a high-risk observational setting that demands exceptional ethical scrutiny, transparency, and long-term accountability. While ethical and regulatory debates dominate public discourse, TEG also presents a research opportunity to systematically evaluate the CV effects of PED use under controlled conditions. A dedicated, risk-adapted pre-participation screening and longitudinal monitoring framework will be essential for characterizing PED-associated CV effects and informing harm-reduction strategies.

## Key Points

1. “The Enhanced Games” is a disruptive sporting event in which pharmacological performance enhancement using FDA-approved drugs is permitted.
2. While PED exposure is associated with potentially relevant cardiovascular risks, current evidence remains limited, heterogeneous, and largely confined to selected substances and populations.
3. Enhanced athletes are not physiologically equivalent to natural athletes, and a dedicated, risk-adapted cardiovascular pre-participation screening with longitudinal follow-up is essential to characterize risk and support harm-reduction strategies.

## Background

The Enhanced Games (TEG) is a controversial initiative conceived to revolutionize competitive sports and push the boundaries of human physical performance. A key element of TEG is the optional use of performance-enhancing drugs (PEDs), disrupting traditional sports culture by challenging the cornerstone of regulated and fair athletic competition. As indicated by the organizers, the project prioritizes athlete-centrism, scientific advancement, the exploration of boundaries in human performance, sustainability, and the financial appreciation of athletes [1]. Notably, the use of illicit drugs will be prohibited; only FDA-approved substances may be taken by athletes. However, it is not clarified whether PEDs will be allowed only during training and preparation, or in the contest as well, or whether any formal antidoping controls will be implemented to enforce the prohibition of illicit substances [1].

The first TEG event is scheduled to take place in May 2026 in Las Vegas, where participants can compete in short-distance swimming, track & field (sprint and hurdles), and weightlifting. According to the initiative’s website, an independent Medical Commission will oversee medical profiling and eligibility and ensure safety, while the Scientific Commission will be tasked with ensuring scientific rigor [1]. On the forefront of TEG are active and former professional athletes looking to push boundaries of human performance and break world records. The number of participants was 50 at the time this manuscript was written.

The announcement of TEG has triggered widespread reactions across the sporting and medical communities, including strong concerns regarding athlete safety, ethics, and the potential normalization of pharmacological enhancement [2]. In this context, TEG has been proposed by organizers as a more transparent competitive environment, contrasting with the pervasive clandestine pursuit of PEDs not yet recognized by the World Anti-Doping Agency (WADA) in mainstream sport, a practice that introduces unfair advantages and poorly characterized health risks [3]. Regardless of these considerations, the TEG framework raises pressing and unresolved questions regarding the short- and long-term health of participating athletes [4].

According to current WADA criteria, a substance may be prohibited if it meets at least two of three conditions: the potential to enhance performance, the potential to pose a health risk to the athlete, or violation of the spirit of sport [5]. As a result, several prohibited agents may be included primarily because of their ergogenic effects and implications for competitive fairness rather than because of clearly established CV or systemic toxicity. This distinction is important when interpreting the evolving regulatory landscape surrounding pharmacological performance enhancement.

This narrative review does not advocate for TEG but aims to critically analyze the cardiovascular (CV) risks and research challenges associated with their proposed framework, where athletes openly prepare and compete using FDA-approved substances for off-label extreme performance enhancement. The scope of this review will be confined to drugs that carry or may carry the most significant CV effects, and it does not discuss common, legal ergogenic dietary supplements. Lastly, although many aspects and narratives have evolved over the last three years of the TEG initiative, we will base our arguments on the information currently published on the official website.

Accordingly, this review has two complementary aims: first, to summarize current evidence regarding the CV effects—both ergogenic and adverse—of major classes of performance-enhancing drugs; and second, to discuss the clinical, methodological, and research implications of these exposures within the emerging context of TEG. Literature was identified through targeted PubMed and Google Scholar searches focusing on performance-enhancing substances and cardiovascular outcomes, prioritizing guidelines, original studies, and consensus statements, supplemented by reference screening and author expertise.

## Cardiovascular Diagnostic Challenges in Athletes With and Without PEDs

The shared mission of sports cardiology is to develop diagnostic, primary, and secondary preventive measures to minimize the risk of sudden cardiac death (SCD) and the burden of CV disease in and out of competition, while enabling athletes to reach their maximal cardiopulmonary potential. As such, the global sports cardiology community has invested significant effort over the last few decades in describing CV adaptation to regular, vigorous physical activity [4, 6, 7].

Increased ventricular wall thickness, chamber enlargement, enhanced stroke volume, and augmented diastolic filling represent adaptive responses to sustained training load and are generally associated with preserved or even supranormal functional reserve. Consequently, some structural and functional findings reported in studies of athletes using PEDs may overlap with features of the so-called “athlete’s heart” and should not be automatically regarded as pathological [8, 9]. The coexistence of pharmacological exposure introduces additional diagnostic complexity, as it may blur the boundary between physiological adaptation, transient remodeling, and early cardiotoxic effects. Indeed, the complexity and dynamics of cardiac adaptation, along with overlapping features with certain heart diseases (e.g., dilated cardiomyopathy, hypertrophic cardiomyopathy, and beyond), pose significant challenges for developing robust, diagnostic, and prognostic criteria and distinguishing healthy adaptation from pathology [8, 10]. There is ongoing debate about the length, vigor, and quality of sports activity, which may be detrimental to the CV system in the long term [9]. Moreover, an individualized approach is imperative in the comprehensive CV pre-participation screening (PPS) of athletes, emphasizing the implications of sex, age, race, risk factors, and sports type [7]. The addition of PEDs to the equation introduces additional confounding, which can be challenging to adjust for in clinical and scientific settings.

In practice, athletes may legally use substances not yet included on the WADA Prohibited List, despite limited safety data [3]. Regulatory inertia can allow such practices to spread before adequate evidence becomes available. While the prevalence of prohibited PED use is lower or harder to quantify in elite sport, exposure remains common in recreational and sub-elite athletes and represents a relevant clinical concern [11]. Studying such exposure in transparent and well-monitored settings may help characterize risks rather than imply endorsement. In recent years, the wide availability due to loosened regulations around online prescription and consequent rapid spread of compounded drugs sold by trending telehealth companies have made PEDs more accessible than ever for recreational users pursuing performance-enhancing or general well-being benefits [12]. A recent report estimates that the global subscription-based market for testosterone supplementation has reached $1.08B in 2024 [13]. Despite ongoing controversy surrounding the use of anabolic-androgenic steroids (AAS), growth hormone (GH) derivatives, and other PEDs, the medical community must acknowledge their increasing prevalence in the general population and be prepared to provide informed, evidence-based care to individuals exposed to these substances.

## Performance-Enhancing Drugs and CV Effects

The following sections primarily focus on the CV physiological and pathophysiological effects of selected PED classes, providing the biological and clinical foundation necessary to interpret the potential health implications of pharmacologically enhanced competition.

A body of case reports and small series has reported the cardiac effects of PEDs that provide a basis for the unfavorable perception of many of these drugs [14–19]. Notably, large-scale data among male and female bodybuilders have recently called for action and enhanced preventive measures against SCD [20, 21]. However, large-scale registry data remain scarce and often underrepresent most athletic subpopulations, making extrapolation of many findings unfeasible [22]. Most studies report on AAS, GH, erythropoiesis-stimulating agents (ESAs), and stimulants in the non-athlete population. Other significant sources of confounding include co-morbidities and the practice of “stacking,” or polypharmacy (i.e., the simultaneous or sequential usage of multiple enhancing substances and even illicit drugs in some groups), which raise significant hurdles in differentiating and weighing the effects of respective agents. Another major methodological limitation across the existing literature is the lack of detailed exposure characterization, including precise identification of specific compounds, cumulative dosage, duration of use, and patterns. Consequently, most available studies demonstrate associations rather than causal relationships, and the magnitude of CV risk remains difficult to quantify with precision. Furthermore, many cohorts are derived from highly selected recreational strength-training populations or self-referred clinical samples, plausibly introducing selection and publication bias.

**Fig. 1:**
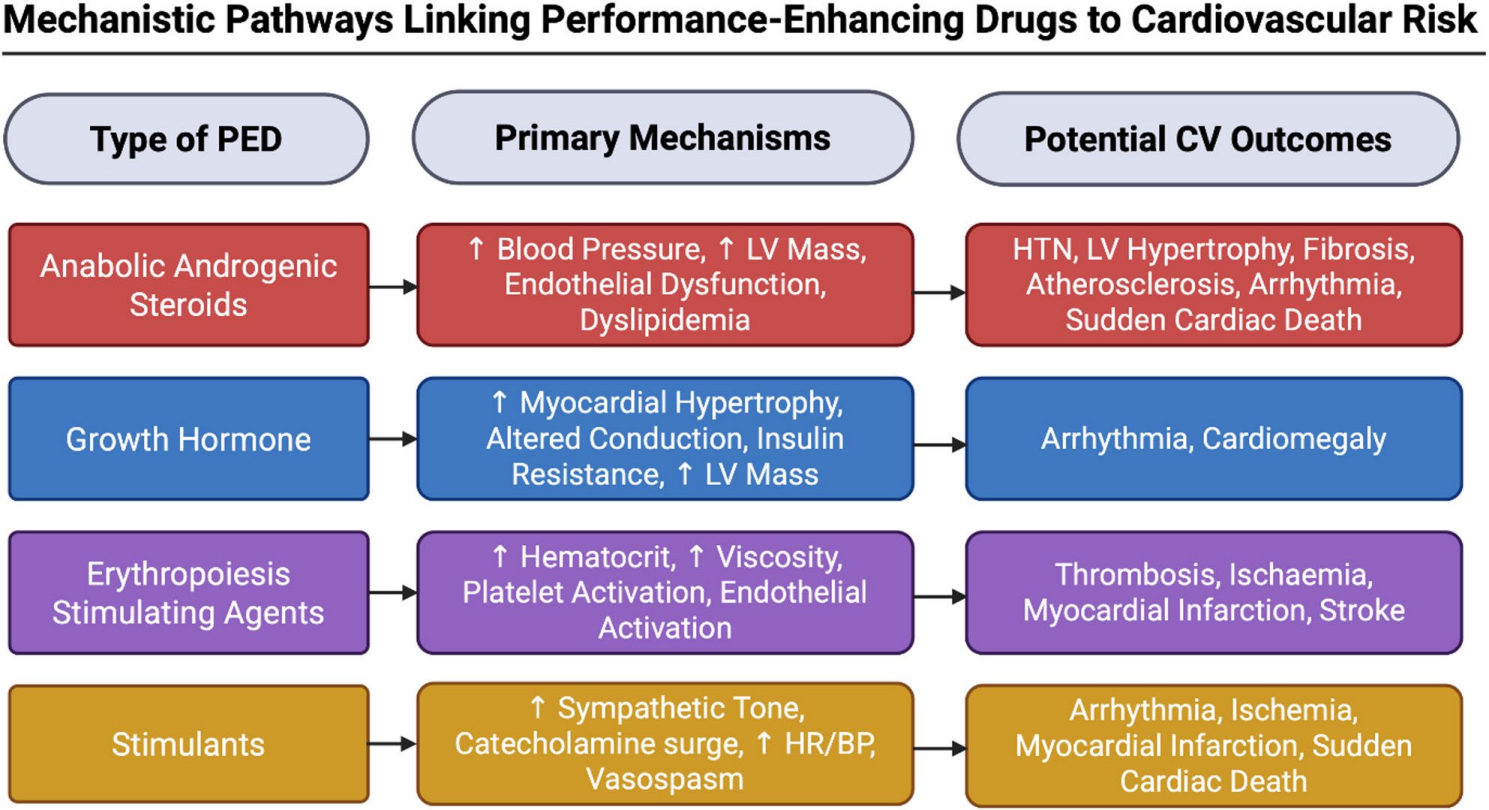
Associations between the most used PEDs and potential CV outcomes. This figure focuses on selected PED classes with established cardiovascular mechanisms and is not intended to be exhaustive. BP = Blood Pressure; CV = Cardiovascular; HR = Heart Rate; HTN = Hypertension; LV = Left Ventricular; PED = Performance-Enhancing Drugs. Created using BioRender.com

Below, we focus on high-quality evidence regarding the most commonly (ab)used FDA-approved agents that TEG participants will ostensibly use and the primary mechanisms that may lead to adverse CV outcomes (Fig. 1).

### Anabolic Androgenic Steroids

AAS exert their ergogenic effects primarily through androgen receptor activation in skeletal muscle, leading to enhanced protein synthesis, nitrogen retention, and inhibition of catabolic pathways. These mechanisms promote marked increases in muscle mass, strength, and endurance, particularly when combined with resistance training [23–25].

While these effects are performance-enhancing, their ergogenic benefits are counterbalanced by plausible CV risks. In a recent Danish nationwide registry, long-term AAS users exhibited over a threefold higher risk of myocardial infarction (MI) and coronary revascularization and a 3.6-fold higher risk of heart failure (HF) over an average follow-up of 11 years [26]. In the same cohort, all-cause mortality due to natural (HR 2.24) vs. unnatural (HR 3.64) causes was significantly higher in AAS users than age-matched controls [27]. However, participants were all recreational athletes, and AAS usage length and dose data were unavailable [26, 27].

The HAARLEM prospective study evaluated adult male fitness center visitors intending to start an AAS cycle and followed them over 1 year [28]. Multiple time-point measurements with 3D echocardiography revealed a 5% decline in left ventricular ejection fraction (LVEF), a drop of −0.45 in the E/A ratio, and an increase in left atrial volume after a median of 16-week-long AAS cycle. Left ventricular mass also increased by an average of 28 g, indicating significant positive associations with AAS dosage. Notably, all imaging parameters normalized after a recovery period of a median of 8 months [29].

Baggish et al. recruited 140 male weightlifters (34–54 years old), of whom 86 had a history of AAS use for at least 2 years, and 54 were non-users. In a cross-sectional cohort design, transthoracic echocardiography (TTE) and coronary computed tomography angiography (CCTA) were employed. Long-term AAS users showed reduced LVEF (mean 49% in users vs. 58% in non-users), LV diastolic function, and higher coronary artery plaque volume than non-users. Moreover, lifetime AAS dose was associated with a higher coronary atherosclerotic burden [30]. In another cross-sectional, single-center study with 101 weightlifter AAS users and 71 non-users, TTE confirmed higher LV mass index, lower LVEF, lower right ventricular global longitudinal strain, and higher systolic blood pressure in AAS users of > 1 year of cumulative AAS use compared to non-users. The study reported a lasting lower-than-normal LVEF in former users as well [31]. In a cardiac magnetic resonance (CMR) imaging study involving male subjects aged 18–40, Luijkx et al. reported different cardiac dimensions, biventricular systolic function, and impaired ventricular inflow for AAS users when compared to non-athletes and non-user athletes [32]. Finally, 90 men (32 with current, 31 with former AAS use, and 27 controls) were included in a positron emission tomography/computed tomography (PET/CT) study to assess coronary microcirculation using myocardial flow reserve (MFR). Impaired MFR was observed in 19% of current and 3% of former users, whereas controls showed no impairment. Subclinical impairment of MFR was present in 28% of current users and 26% of former users, but in only 4% of controls [33].

Recent imaging-based studies provide converging evidence that chronic AAS use is associated with adverse cardiac remodeling and subclinical coronary pathology. Cross-sectional data in recreational athletes demonstrate a dose- and duration-dependent association between lifetime AAS exposure and coronary artery calcification, impaired biventricular strain, increased ventricular mass, and reduced left ventricular ejection fraction. Longitudinal evidence further suggests that sustained AAS use is linked to persistent myocardial dysfunction over time, whereas discontinuation may allow partial regression of hypertrophy and functional recovery, highlighting both cumulative cardiotoxicity and incomplete reversibility [34, 35]. Collectively, these studies link chronic AAS use to structural, coronary, and microvascular cardiac injury. Interestingly, recent CMR data suggest that pharmacological testosterone deprivation may also be associated with adverse myocardial remodeling among transgender individuals, highlighting the nuanced role of sex hormone balance in myocardial integrity [36].

Nevertheless, confounding factors—training intensity, concurrent stimulant use, and unstandardized dosing—limit definitive causal inference. Most data derive from male recreational strength athletes, and the literature remains sparse for female users or elite competitors.

#### Research Gaps and Future Directions

Future investigations should apply CMR with T1/T2 mapping and extracellular volume (ECV) quantification to characterize diffuse myocardial fibrosis and remodeling patterns in active versus former AAS users. Longitudinal, multi-parametric CMR studies incorporating myocardial strain and sex-specific analysis could clarify the reversibility, temporal evolution, and clinical significance of AAS-related cardiotoxicity. Myocardial scar (focal or diffuse) formation can have significant arrhythmogenic potential, necessitating rigorous, longitudinal rhythm monitoring in affected athletes.

### Androgen (Post-)Cycle Supplementation

Androgen cycle supplementation represents a set of adjunctive pharmacologic strategies designed to mitigate the adverse effects of AAS and to preserve endogenous hormonal axes and fertility [37, 38]. Commonly, these regimens involve co-administration of non-AAS hormonal agents. For these purposes, human chorionic gonadotropin (hCG) may be used to counteract AAS-induced testicular atrophy, impaired spermatogenesis, and suppression of gonadotropins [37, 38]. In a pediatric cohort, hCG treatment has been associated with increased LV mass index in cryptorchid boys, likely mediated by elevated circulating testosterone [39].

Selective estrogen receptor modulators (SERMs) – notably tamoxifen and clomiphene - may be used by males taking AAS to antagonize estrogen’s effects in peripheral tissues, such as preventing gynecomastia [40]. While SERMs reduce receptor activity, they do not lower circulating estrogen levels; clomiphene, in particular, can increase serum estrogen indirectly through gonadotropin stimulation [40]. Tamoxifen has been associated with increased venous thromboembolic risk (VTE) in males treated for breast cancer, while the CV effects in women are mixed and context-dependent [41, 42]. Although these agents are widely used during AAS “post-cycle therapy,” there are no controlled data in AAS users regarding CV outcomes. Clomiphene may be used off-label to increase testosterone levels in male hypogonadism and to restore the hypothalamic–pituitary–gonadal axis, which may also exert a beneficial effect on lipid profiles [43]. Notably, pulmonary embolism has been reported as a rare complication of clomiphene therapy [44].

Aromatase inhibitors (AIs)—anastrozole, letrozole, exemestane—reduce the peripheral conversion of testosterone to estrogen. However, large epidemiologic studies in women with breast cancer show higher rates of heart failure and cardiovascular mortality with AIs compared with tamoxifen [45, 46]. In men, AIs can normalize serum testosterone in obesity-related hypogonadism; however, randomized data on CV safety are lacking, and some reports indicate decreased insulin sensitivity in otherwise healthy males [47, 48, 49].

Exogenous AAS can also increase prolactin levels, leading to gynecomastia, decreased libido, and erectile dysfunction in males [50]. Dopamine agonists, such as cabergoline or bromocriptine, may help counteract these effects but are associated with a higher prevalence of tricuspid regurgitation [51]. 5-α-reductase inhibitors (e.g., finasteride, dutasteride) may be used to prevent androgen-related alopecia during AAS cycles; however, they can also further disrupt androgen signaling. Additionally, they have been associated with sexual dysfunction, neuropsychiatric, and metabolic effects, although clinical CV data remain scarce. Phosphodiesterase-5 inhibitors are co-used to counteract AAS-related erectile dysfunction; although generally considered safe, their chronic use in the context of supraphysiologic androgen exposure and cardiac remodeling has not been systematically studied.

#### Research Gaps and Future Directions

Altogether, AAS cycles and the accompanying supplementation agents form a complex pharmacologic regimen with largely undocumented CV consequences beyond those directly attributed to AAS themselves. Current evidence is confined to isolated case reports, extrapolated data from endocrine disorders, and small non-athlete cohorts. High-quality rhythm monitoring data and tissue-level insights—especially myocardial characterization using advanced CMR—are virtually absent. Quantitative T1 and ECV mapping could elucidate diffuse myocardial fibrosis in AAS users and individuals engaging in hormonal cycle therapies, a pathophysiologic substrate strongly linked to adverse outcomes and HF risk [52, 53].

### Growth Hormone and Insulin-Like Growth Factor-1 (IGF-1)

GH and its downstream effector, insulin-like growth factor-1 (IGF-1), have become popular agents among athletes aiming to enhance lean body mass, accelerate recovery, and improve exercise performance—often in combination with insulin or anabolic steroids. However, evidence for true ergogenic benefit remains limited. At supraphysiologic levels, GH and IGF-1 promote sodium and water retention, sympathetic activation, and myocardial hypertrophy, establishing a plausible biological link to hypertension, arrhythmia, and cardiomyopathy [54–56].

Randomized controlled trials and systematic reviews in healthy adults showed modest gains in lean body mass without improvements in strength or exercise outcomes, while adverse effects such as edema, fatigue, arthralgias, and impaired glucose tolerance were frequent [57, 58]. Insights from acromegaly further support this concern: patients with acromegaly, characterized by chronic GH/IGF-1 excess, may develop LV hypertrophy, diastolic dysfunction, and arrhythmias, with partial reversibility after biochemical control [54, 59, 60].

Large registry studies in childhood GH recipients have reported increased risks of CV disease and hemorrhagic stroke, with higher risks at longer and higher cumulative exposures [61–63]. Although these findings derive from replacement therapy in deficiency or growth-disorder contexts, rather than enhancement, they highlight potential dose–duration relationships. Conversely, observational data in adults with GH deficiency demonstrate neutral or favorable cardiovascular risk profiles when GH is replaced at physiological doses [59, 60, 64].

Direct evidence among athletes is limited. Case reports describe severe dilated cardiomyopathy in bodybuilders using GH and IGF-1, typically in combination with anabolic steroids [15]. Narrative and mechanistic reviews emphasize that detection of GH/IGF-1 misuse remains difficult, co-use of other performance-enhancing drugs obscures attribution, and no prospective athlete cohorts exist to quantify long-term CV risk [54, 65]. While performance benefits are dubious, it is biologically plausible that prolonged, supraphysiologic GH/IGF-1 exposure is associated with increased cardiometabolic and myocardial structural risk, likely mediated by increased wall thickness and the promotion of myocardial fibrosis, particularly in polypharmacy contexts [66].

Overall, GH and IGF-1 misuse is biologically plausible as a cause of hypertension, arrhythmia, and cardiomyopathy, but direct evidence in athletes is sparse. Clinical trials in healthy adults, however, show no true performance benefit but frequent adverse effects [57, 58]. Registry data from childhood GH exposure suggest higher CV risk, whereas adult replacement therapy appears neutral at physiological doses [59–64]. Case reports and the acromegaly model illustrate potential for severe cardiomyopathy [15, 54, 55].

#### Research Gaps and Future Directions

Systematic cardiac assessment in GH/IGF-1 users remains lacking. Advanced imaging, particularly CMR with T1/T2 mapping and ECV quantification, could clarify whether diffuse myocardial fibrosis or subclinical remodeling parallels the cardiomyopathy/cardiomegaly observed in acromegaly. Prospective, longitudinal studies integrating rhythm monitoring, imaging, blood biomarkers, and dose-response modeling would be critical to disentangle physiological adaptation from early cardiotoxicity in users of enhancement and those seeking well-being effects.

### Erythropoiesis-Stimulating Agents and Blood Doping

Recombinant human erythropoietin (EPO), darbepoetin, and other ESAs have been widely misused in endurance sports to increase hemoglobin concentration, enhance oxygen delivery, and thereby improve aerobic performance [67, 68]. Emerging evidence continues to document both the prevalence and physiological consequences of blood manipulation. A global analysis of elite track and field athletes estimated blood doping prevalence around 18% in 2011 and 15% in 2013 [69]. More recently, a survey of U.S. elite athletes reported that 0.6% admitted to using blood manipulation [70]. Recent reviews further emphasize the increasing use of micro-dosing strategies, where small, repeated ESA administrations or transfusions are employed to achieve performance benefits while minimizing hematological fluctuations [71].

Similar physiological effects can be achieved through autologous or homologous blood transfusions (“blood doping”). While these interventions effectively augment maximal oxygen uptake and endurance capacity, they may also carry substantial CV risk [72, 73]. The primary mechanism is a rise in hematocrit and whole-blood viscosity, predisposing to hypertension, arterial and venous thrombosis, and arrhythmia [74, 75]. Secondary pathways include endothelial dysfunction, impaired nitric oxide bioavailability, and platelet hyperreactivity, creating a pro-thrombotic milieu [76, 77].

Large, randomized trials in non-athletic populations have demonstrated safety concerns associated with certain ESAs. In the CHOIR trial, patients with chronic kidney disease (CKD) randomized to a higher hemoglobin target with epoetin alfa experienced significantly more death, acute MI, congestive HF, and stroke [80]. Similarly, the TREAT trial of darbepoetin alfa in 4,038 diabetic CKD patients with anemia showed nearly doubled stroke risk without outcome benefit [81]. Although conducted in clinical rather than athletic populations, these findings highlight the potential vascular risk of pharmacologically elevating hemoglobin.

Historical reports from elite endurance sports in the late 1980 s and early 1990 s describe clusters of sudden deaths—particularly among professional cyclists—strongly suspected to involve EPO misuse, often compounded by dehydration and intense training [78]. The risk appears continuous rather than threshold-based: population studies reveal progressively higher thrombotic and hypertensive risk with rising hematocrit, even below the 50% level historically adopted as a regulatory cutoff [79–81]. Experimental and athlete-based data indicate that blood manipulation strategies can induce transient elevations in hematocrit to values exceeding 50%, a range particularly hazardous when combined with dehydration or heat stress [75, 79–84]. From a clinical and regulatory perspective, no safe threshold for ESA or transfusion misuse can be defined.

In summary, ESAs and blood transfusions can reliably enhance endurance capacity by raising hemoglobin mass, but this is likely accompanied by an increased CV risk in select populations. Large-scale studies suggest higher rates of stroke, MI, HF, and death with hematocrit elevation in non-athletes [79, 85, 86]. Importantly, even with micro-dosing, cumulative increases in hemoglobin mass and viscosity persist, and adverse outcomes remain plausible.

#### Research Gaps and Future Directions

Direct CV phenotyping of ESA and blood-doping users remains absent. Future work should leverage CMR with parametric mapping and aortic flow analysis to investigate subclinical changes in myocardial function and vascular stiffness associated with repeated erythropoietic stimulation. Longitudinal athlete registries combining imaging, rhythm monitoring and hematologic data, and biomarker profiles could clarify thresholds for reversible versus permanent CV damage.

### Stimulants

FDA-approved stimulant medications relevant to TEG preparation and competition may include amphetamine derivatives (mixed amphetamine salts, dextroamphetamine, lisdexamfetamine), methylphenidate, metamphetamine, and the wake-promoting agents modafinil/armodafinil. These drugs primarily enhance central dopaminergic and noradrenergic signaling by blocking reuptake transporters and facilitating neurotransmitter release, leading to increased alertness, attention, and fatigue resistance, and appear to have an overall positive effect on athletic performance [87].

A recent meta-analysis including 93 randomized trials with stimulants reported an increased risk of overall adverse events, compared to placebo. However, the pooled data on CV adverse events only suggested higher diastolic blood pressure and heart rate without apparent clinical relevance [88]. In attention-deficit/hyperactivity disorder populations, meta-analyses and large registry-based studies consistently demonstrate modest, dose-dependent increases in heart rate (≈ 5–10 bpm) and systolic blood pressure (≈ 2–5 mm Hg) with amphetamine or methylphenidate use, but no clear increase in major adverse cardiovascular events in otherwise healthy children and adults [89–91]. Nevertheless, rare serious events such as MI, arrhythmia, and SCD have been reported, particularly in individuals with underlying heart disease. In some SCD cases, autopsy confirmed undiagnosed HCM [92].

For modafinil and armodafinil, clinical trials in narcolepsy and sleep-disorder populations generally report minimal average hemodynamic changes, though isolated cases of hypertension and arrhythmia have been described [93]. Pharmacovigilance data from the US suggested no increased risk of MI and CV hospitalization with modafinil [94].

Collectively, the CV evidence base with stimulants shows modest but reproducible hemodynamic effects in young populations, contrasted with serious adverse outcomes reported mainly in case reports and misuse settings. Whether FDA-approved stimulants carry long-term CV risk as part of a performance enhancement regime is unknown.

#### Research Gaps and Future Directions

Despite extensive safety data in ADHD and sleep-disorder populations, the long-term cardiovascular impact of stimulant use for performance enhancement remains unknown. It is currently unknown how stimulants may affect arrhythmic susceptibility and specifically, the incidence of malignant arrhythmias in athletes. Controlled trials in athletic settings are absent, and no imaging or rhythm monitoring data exist to define potential subclinical effects. Future research incorporating CMR and CCTA could clarify whether repetitive sympathomimetic exposure contributes to (subclinical) coronary artery, myocardial, or large vessel remodeling in athletes.

### Inhaled Beta-Agonists

Inhaled β₂-adrenergic agonists such as albuterol/salbutamol, formoterol, and salmeterol are FDA-approved for asthma and chronic obstructive pulmonary disease (COPD). These agents act by selectively stimulating β₂ receptors in airway smooth muscle to promote bronchodilation, while higher systemic exposures may induce lipolysis, glycogenolysis, and potential anabolic effects.

Evidence from randomized trials and meta-analyses in athletes and asthma populations indicates that inhaled β₂-agonists at therapeutic doses improve pulmonary function but do not substantially enhance endurance or strength performance beyond placebo [95, 96]. By contrast, a different meta-analysis reports small gains in sprint/strength (≈ 5% overall) driven by oral or supratherapeutic regimens, while these effects were not observed at WADA-permitted inhaled doses [5, 96, 97]. In a double-blinded, balanced four-way block crossover trial including 24 (12 women) competitive endurance athletes, no ergogenic benefit was observed at the permitted dosages [98].

Regarding CV safety, large observational studies in COPD find no increased MI risk with short-acting inhaled β-agonists (SABA), whereas new initiation of long-acting β-agonists (LABA) has been linked to a ~ 1.5-fold increase in severe cardiovascular events within 30 days [99, 100]. In healthy endurance athletes, acute administration of inhaled salbutamol/formoterol altered LV physiology after a 10-minute cycling session, yet no serious CV events were observed [101].

At the case level, reports describe supraventricular tachycardia after SABA treatment in children, QT prolongation, Takotsubo cardiomyopathy, and acute MI following excessive albuterol/salbutamol exposure in an elderly patient, highlighting potential risks with high doses or in vulnerable individuals [102–106]. In contrast, athlete case reports of cardiovascular injury predominantly involve β₂-agonist misuse/overdose or non-FDA agents, especially clenbuterol, with documented myocarditis and myocardial ischemia/infarction in bodybuilders and a collegiate football player, and ventricular arrhythmias reported after extreme salbutamol exposures [16, 17, 107].

In summary, inhaled β₂-agonists at approved therapeutic doses improve airway function and exercise tolerance in asthmatic or trained individuals without consistent evidence of ergogenic benefit or major cardiovascular harm. However, misuse involving high-dose or oral administration and non-approved β₂-agonists (e.g., clenbuterol) carries clear arrhythmic and ischemic risk.

#### Research Gaps and Future Directions

No prospective studies have quantified cardiovascular remodeling or arrhythmia burden in long-term β₂-agonist user athletes. Future research employing advanced imaging with CMR, CCTA, and rhythm monitoring could clarify whether sustained β₂-receptor stimulation or polypharmacy contributes to (subclinical) myocardial or vascular injury.

### New Weight-Management Agents

While unlikely to be an integral part of performance-enhancing regimens in TEG participants, novel weight-loss agents have been widely recognized for their beneficial CV effects across various patient populations. Notably, glucagon-like peptide 1 (GLP-1) receptor agonists (semaglutide, liraglutide) and the dual gastric inhibitory peptide (GIP)/GLP-1 agonist tirzepatide promote weight loss primarily via hypothalamic appetite regulation and delayed gastric emptying (with GLP-1), with tirzepatide adding GIP-mediated effects on adiposity and insulin sensitivity [108–111].

Significant cardiovascular outcomes trials in patients with obesity or diabetes consistently demonstrate reduced major adverse cardiovascular events (MACE) with GLP-1 receptor agonists, including semaglutide and liraglutide, and a neutral-to-beneficial profile for tirzepatide in early analyses [112]. These cardioprotective effects appear mediated by weight reduction, improved endothelial function, and anti-inflammatory actions [108–111].

For athletes, these drugs may act as indirect performance enhancers, conferring body-composition or weight-class advantages in select sports. Notably, GLP-1 analogues have recently gained traction among bodybuilders [113]. However, their use in healthy or athletic individuals has not been tested in randomized trials, and the long-term cardiovascular implications in this context remain unknown. In contrast, stimulant-like anorectic agents—sometimes co-opted for rapid weight loss—require close hemodynamic monitoring due to sympathetic activation and potential arrhythmic risk.

Prospective data in athletes are needed to elucidate how GLP-1 and GIP/GLP-1 agonists affect cardiac structure, function, and cardiopulmonary performance.

### Other Agents—Diuretics, Thyroxin, Insulin, Trimetazidine

Diuretics, including loop, thiazide, and potassium-sparing agents, have a significant history of misuse in aesthetic or weight-class sports to achieve rapid weight loss or mask other PEDs [114, 115]. Their mechanism involves natriuresis and plasma volume reduction, resulting in transient weight loss without a true ergogenic benefit. However, misuse can provoke dehydration, electrolyte imbalances (notably hypokalemia and hyponatremia), QT prolongation, and ventricular arrhythmias, occasionally leading to sudden cardiac arrest [116, 117]. Case evidence describes severe arrhythmia following indapamide-induced hyponatremia and hypokalemia, and a higher prevalence of minor exercise-induced arrhythmias (isolated premature atrial and ventricular complexes) among diuretic users compared to controls [118, 119]. Reviews of diuretic abuse in sports emphasize their masking potential and cardiac risks due to electrolyte shifts and dehydration, and a fatal case of torsemide misuse has been reported [120, 121].

Thyroxine (levothyroxine) is FDA-approved for hypothyroidism and thyroid cancer suppression, acting as a synthetic T4 hormone; in euthyroid athletes, supraphysiologic doses can promote weight loss and heightened metabolic rate, but carry risks of atrial fibrillation, tachyarrhythmia, and bone loss [122]. Observational data in patients with suppressed TSH due to overtreatment demonstrate increased risks of atrial fibrillation and other adverse CV outcomes [123].

Insulin, approved for the treatment of diabetes mellitus, facilitates glucose uptake and anabolic processes; in bodybuilding settings, it has been misused to augment glycogen and muscle protein accretion [124]. While controlled trials in athletes are lacking, reviews highlight its reputation as one of the most dangerous PEDs due to the high risk of severe hypoglycemia, arrhythmias, seizures, coma, and death [125]. Cases of non-diabetic individuals hospitalized after insulin misuse confirm the potential for life-threatening neuroglycopenia and arrhythmias [126].

Trimetazidine is a metabolic modulator used for angina and HF in parts of Europe and Asia, but it is not FDA-approved. Clinical trials in patients with ischemic heart disease show improvements in exercise tolerance and a decrease in angina frequency [127]. While CV adverse events are uncharacteristic, a systematic review notes multiple case reports of trimetazidine-induced reversible Parkinsonism (rigidity, akinesia, gait disturbance) that resolved after drug withdrawal [128].

In summary, these agents, though mechanistically diverse, illustrate how drugs outside the classical performance-enhancing spectrum can be repurposed or misused for metabolic or conditioning purposes, often with disproportionate CV or neurological risks.

## Future Research Directions in Enhanced Athletes

The agents outlined above represent only a subset of drugs with established and/or putative positive ergogenic and CV adverse effects (Table 1). Yet, systematic, prospective research on the CV impact of traditional PEDs remains lacking, underscoring a major evidence gap in both sports medicine and preventive cardiology. Establishing international registries that incorporate standardized cardiac imaging, circulating biomarkers, and long-term clinical outcomes could refine risk assessment frameworks and inform both regulatory and preventive strategies. Importantly, most existing data are derived from young male strength training cohorts; women and non-White athletes remain markedly underrepresented, limiting generalizability and obscuring potential sex- and ethnicity-specific effects.

**Table 1:** Summary of the effects of the drugs most used for off-label performance enhancement

PED class	Primary mechanism	Ergogenic effect	Typical athletic use	Short-term effects	Long-term effects	Athlete-specific evidence	Level of evidence		References
							Ergogenicity	Risk	
AAS	Androgen receptor activation → ↑ protein synthesis, myocyte hypertrophy; adverse lipid and coagulation shifts	↑ Strength and fat-free mass; possible power- gains	Bulking/cutting cycles, strength and physique sports (e.g., body building)	↑ BP, mood changes; ↑ Hct; ↓ HDL/↑ LDL	LVH/LV dysfunction, arrhythmias, CM, MI; coronary microvascular dysfunction; ↑ mortality signal	CMR/echo - ventricular dysfunction; case series report SCD in strength athletes	High	High	[14, 18, 19, 23–35, 37]
Growth hormone/IGF-1	GH/IGF1 signaling → fluid retention, soft tissue- and myocardial hypertrophy; insulin resistance	Small or uncertain effects on sprint power; minimal effect on strength/endurance	Recovery, leanness, synergy with AAS	Edema, arthralgia, paresthesia; glycemic perturbation	Historical acromegaly cardiomyopathy; modern AGHD replacement cohorts show mixed CV risk; pediatric GH - late CV events in some cohorts	No athlete RCTs showing clear performance benefit; observational athlete data sparse	Low–Moderate	Moderate (replacement data)	[15, 54–66]
ESAs and blood manipulation	↑ RBC mass/hematocrit → ↑ O2 delivery; ↑ viscosity; platelet activation	↑ VO_2_max and endurance performance; large effect with autologous transfusion	Endurance sports; altitude simulation; transfusion before events	HTN, headaches; hyperviscosity symptoms	↑ VTE, stroke, and CV events at higher Hb targets (CKD trials); thrombotic risk	Transfusion trials show performance gains; prevalence studies indicate ongoing use	High	High	[67–86]
Stimulants	↑ Central/peripheral catecholamines → ↑ arousal, HR, BP	Small improvements in vigilance and time-to-exhaustion in some settings	Focus, reaction time, fatigue resistance	Tachycardia, BP ↑, insomnia, anxiety	Arrhythmias and ischemia are rare; population data show low absolute CV risk with ADHD doses	Meta-analyses show mixed performance effects; no clear athlete CV excess at therapeutic doses	Moderate	Low–Moderate (therapeutic); higher with misuse	[87–94]
β2-agonists and clenbuterol (non-FDA approved)	β2 stimulation → bronchodilation; systemic use → chronotropy, hypokalemia	Minimal with non-asthmatics by metanalyses; clenbuterol evidence poor	Asthma therapy within thresholds; off label- fat loss (clenbuterol)	Tremor, tachycardia; SVT and QT prolongation reported	Case reports of MI, Takotsubo; myocarditis with clenbuterol	RCTs show little benefit on TT performance; case reports in athletes describe arrhythmias/MI	Low (inhaled in non-asthmatics)	Low–Moderate overall; higher with supra-therapeutic/clenbuterol	[5,16,95–107]
Novel weight management agents	GLP-1/GIP agonism → weight loss and cardiometabolic improvements	No established ergogenic effect	Body composition and weight control	GI symptoms; reduced appetite	Reduced MACE in obesity/T2D trials	Nonspecific; extrapolated from CV outcome trials	Very low	N/A (therapeutic; CV benefit in high-risk- groups)	[108–113]
Diuretics	Natriuresis and volume loss; electrolyte shifts	Weight reduction for weight class- or aesthetics; masking of other agents	Rapid weight cuts; concealment	Hypokalemia/hyponatremia → arrhythmia; dizziness	Arrhythmias and rare fatalities with abuse	Anti-doping literature documents misuse; case reports describe arrhythmias	Low	Moderate	[114–121]
Other: thyroxine, insulin, trimetazidine (non-FDA approved)	Thyroxine: ↑ metabolic rate; Insulin: glucose disposal → hypoglycemia risk; Trimetazidine: FAO inhibition → metabolic shift	Unclear for thyroxine/insulin in healthy athletes; trimetazidine not ergogenic	Leanness (thyroxine), nutrient partitioning (insulin), speculative endurance (trimetazidine)	Tachycardia (thyroxine); hypoglycemia, QT risk (insulin); nausea/dizziness (trimetazidine)	Over-replacement thyroxine linked to CV morbidity; insulin misuse → severe hypoglycemia; trimetazidine → parkinsonism	Mostly absent; evidence from clinical populations and case reports	Very low	Low–Moderate	[122–128]

Systematic assessment of rhythm abnormalities through ambulatory and exercise-triggered ECG monitoring may represent an early, clinically meaningful strategy for detecting electrical instability in pharmacologically enhanced athletes. CMR and blood biomarker-focused studies—particularly using high-sensitivity troponin and natriuretic peptides (BNP, NT-proBNP)—could serve as early, prognostically relevant tools for detecting subclinical myocardial injury, fibrosis, and remodeling. Integrating coronary artery imaging and arrhythmic risk profiling may also refine risk stratification for SCD prevention.

A further unmet question is whether standard CV preventive therapies—including aspirin, statins, beta-blockers, and renin–angiotensin–aldosterone system inhibitors—retain similar efficacy in current or former PED users as in the general population. Even for AAS users, the most studied PEDs group, low evidence yet addresses whether these agents mitigate long-term cardiovascular morbidity or mortality. Emerging therapies with cardiorenal benefits, such as sodium-glucose cotransporter 2 inhibitors, therefore, represent an additional area for future investigation.

Overall, accumulating evidence suggests that several PED-associated CV alterations may be preventable or partially reversible if identified at an early stage. Systematic screening strategies incorporating biomarkers and longitudinal imaging may facilitate timely recognition of myocardial dysfunction and inform clinical decision-making, including cessation of the offending agent, with the potential to mitigate long-term cardiovascular risk.

Future research in pharmacologically enhanced athletes should prioritize the development of prospective cohort studies in clearly defined elite and sub-elite athletic populations, with systematic characterization of PEDs exposure, including compound-specific dosage, duration of use, stacking practices, and timing relative to training cycles. Such designs would allow a more accurate assessment of dose–response relationships and temporal patterns of CV adaptation or injury. Additional priorities include comparative investigations of short-term versus long-term exposure, sex-specific CV effects, and the potential role of genetic and epigenetic mechanisms in modulating individual susceptibility to pharmacologically induced cardiac remodeling [129–131]. Furthermore, the reversibility of myocardial structural and electrical alterations following PED discontinuation remains insufficiently understood and warrants targeted longitudinal investigation.

## Clinical Considerations and Scientific Opportunities

Building upon the pharmacological and CV considerations discussed above, the following section addresses the broader clinical and research implications of PED exposure within the specific framework of TEG.

The CV implications of PED exposure extend well beyond the context of TEG and represent an increasing clinical challenge. An international position statement from the European Association of Preventive Cardiology has identified distinct populations of PED users, highlighting the need for improved understanding of PED-related CV effects and the physician’s role in harm reduction [132]. Within this framework, TEG should not be viewed as a validation model for enhancement, but rather as a high-risk observational setting that may provide structured insights, provided that rigorous PPS, longitudinal monitoring, and cautious interpretation are applied.

### Cardiovascular Pre-Participation Screening in Enhanced Athletes

**Fig. 2:**
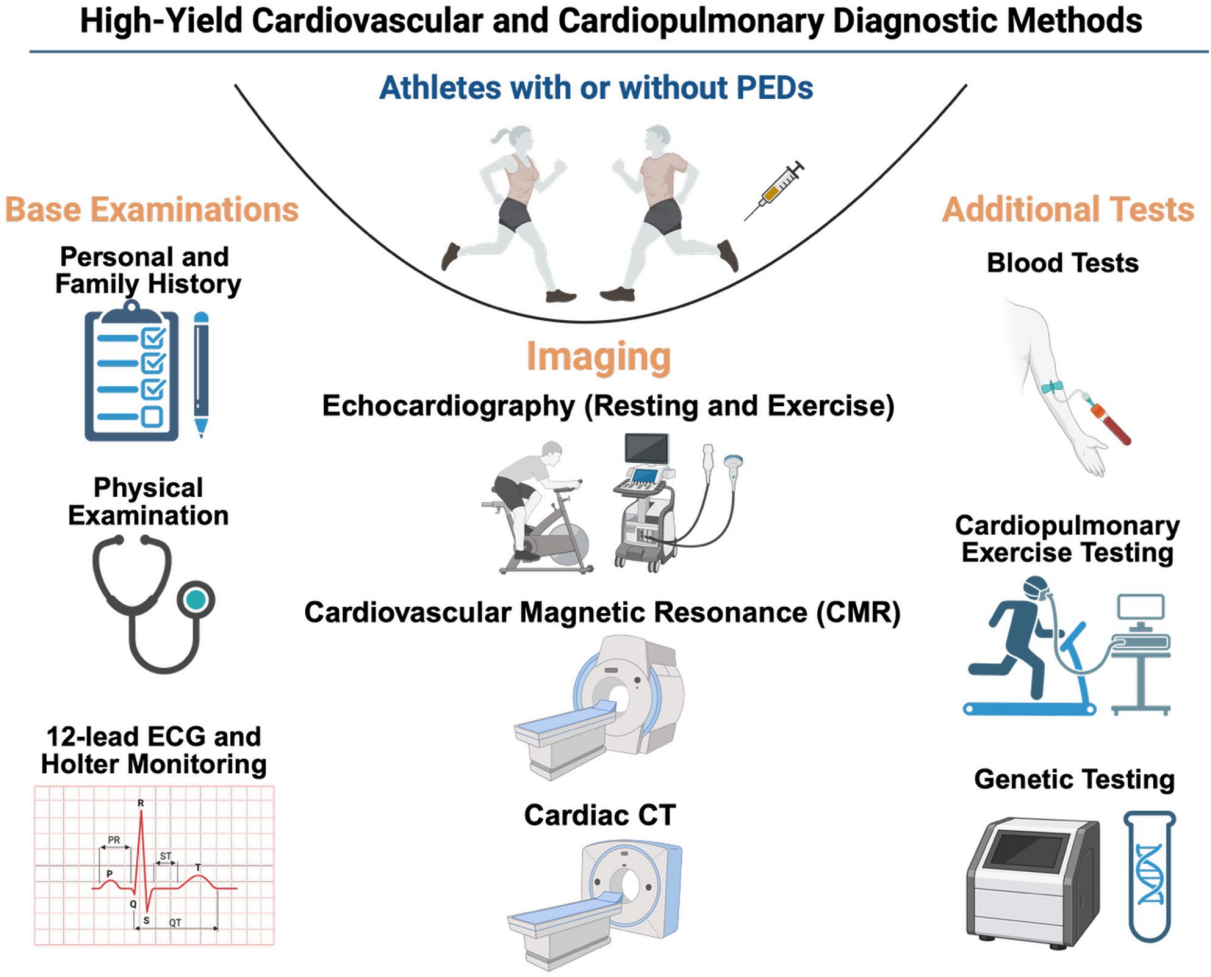
High-yield diagnostic tools and methods for cardiovascular pre-participation screening in athletes. Non-exhaustive compilation of diagnostic methods with the highest yield in TEG and non-TEG athletes. Other tools, such as nuclear imaging, invasive coronary angiography, or invasive electrophysiological studies, can also have implications in selected cases; Created using BioRender.com

Athletes openly exposed to PEDs should not be considered physiologically equivalent to “natural” athletes undergoing standard PPS. Pharmacological enhancement may induce structural, electrical, metabolic, and vascular alterations that differ qualitatively and quantitatively from exercise-induced adaptations alone. Consequently, PPS strategies designed for natural athletes may be insufficient to detect early or subclinical cardiovascular abnormalities in enhanced athletes (Fig. 2).

Mandatory PPS protocols are not globally standardized and typically do not include more than obtaining a personal and family history, as well as a physical examination with or without a 12-lead ECG. Additionally, conflicting data on the utility and yield of different approaches continue to fuel debate on optimal screening methods for both young and master athletes (≥35 years) [4].

A tailored PPS framework for enhanced athletes should build upon standard screening while incorporating additional assessments informed by the known and putative CV effects of PEDs. Core components should include a detailed personal and family history—with specific attention to PED exposure patterns (type, dose, duration, stacking practices)—physical examination, resting blood pressure profiling, and anthropometric assessment. Resting 12-lead ECG remains essential to identify arrhythmias, abnormal voltage patterns, and repolarization changes potentially associated with PED exposure. Exercise testing or cardiopulmonary exercise testing (CPET) with ECG monitoring may further unmask exertion-induced arrhythmias, abnormal blood pressure responses, or ischemic changes. Transthoracic echocardiography plays a central role in assessing left ventricular wall thickness, chamber dimensions, systolic and diastolic function, and myocardial deformation parameters, which may help differentiate physiological adaptation from maladaptive remodeling [133].

In this context, we propose that all athletes with declared PED exposure or with a clinical history strongly suggestive of chronic PED use should undergo a comprehensive CV PPS. In selected individuals, advanced investigations, including CMR, CCTA, extended rhythm monitoring, and comprehensive laboratory testing (for example, complete blood count, cardiac necro enzymes and natriuretic peptides, lipid profile, kidney and liver function tests, coagulation parameters, hormone levels, and urine tests), may be warranted to improve risk stratification [134].

Importantly, while establishing a comprehensive CV baseline prior to PED exposure would be ideal, this will often not be feasible in real-world settings, as many athletes may present after prolonged or intermittent use of PEDs. In such cases, CV evaluation should rely on detailed clinical history, careful assessment of PED exposure patterns, and integration of any previous CV investigations performed before or during earlier stages of training. Serial follow-up assessments may help differentiate potentially reversible pharmacologically induced changes from primary CV disease.

At present, no dedicated evidence-based eligibility thresholds exist for athletes participating in pharmacologically enhanced competitions. Clinical decision-making should therefore be guided by established diagnostic criteria used in sports cardiology for major CV conditions, such as cardiomyopathies, clinically relevant coronary artery disease, or high-risk arrhythmic syndromes [4]. In scenarios where clearly pathological findings are identified, including severe ventricular hypertrophy, significant ventricular dilation with functional impairment, substantial coronary abnormalities, or complex ventricular arrhythmias, temporary restriction from competitive participation should be considered according to current sports cardiology principles until clinical stability or evidence of reversibility is demonstrated.

Conversely, the interpretation of borderline or “grey-zone” findings represents a major clinical challenge in enhanced athletes. Mild increases in left ventricular wall thickness, modest chamber enlargement, or isolated premature ventricular beats may reflect a multifactorial interplay between physiological training adaptation, pharmacologically mediated remodeling, and early pathological processes. In the absence of validated PED-specific cut-offs, such findings should not automatically lead to exclusion from competition but rather prompt individualized risk stratification integrating symptoms, family history, exercise response, arrhythmic burden, and longitudinal imaging data when available.

Particular attention should be paid to arrhythmic manifestations, especially exercise-induced, repetitive, polymorphic, or complex ventricular arrhythmias, which may represent markers of underlying myocardial fibrosis or electrical instability [135–137]. Even in the absence of a formally diagnosed cardiomyopathy, these findings may warrant temporary restriction from high-intensity competitive participation due to the potential risk of malignant arrhythmic events, especially in pharmacologically enhanced subgroups.

Overall, while defining eligibility in clearly high-risk clinical scenarios may be relatively straightforward, determining the prognostic significance of milder structural or electrical abnormalities remains considerably more uncertain. For this reason, a reasonably comprehensive and systematically applied CV screening strategy, balanced against cost-effectiveness considerations, appears justified in athletes with known or suspected PED exposure, with eligibility decisions guided primarily by individualized clinical judgement and longitudinal reassessment. Crucially, individuals with pre-existing high-risk CV conditions should be systematically excluded from participation, particularly when strong mechanistic or epidemiological links exist between specific PEDs and CV pathology.

### TEG as an Observational Research Setting

While the permissiveness of PED use inevitably introduces additional health risks, the existence of a controlled and closely supervised environment may allow for systematic observation of short-, mid, and long-term physiological consequences that remain poorly characterized. In this context, TEG could serve as an observational framework, rather than a validation model, for studying the cardiovascular, musculoskeletal, endocrine, and neuropsychological effects of PEDs under stringent medical oversight. A multidisciplinary team including cardiologists, orthopedists, endocrinologists, internists, neurologists, radiologists, and mental health professionals would be essential to ensure rigorous risk assessment, harm minimization, and ethical governance within such a setting. Nevertheless, even advanced medical supervision cannot eliminate the intrinsic risks associated with PED exposure.

Importantly, TEG cannot be regarded as an experimental or interventional model. The absence of randomization, standardized exposure protocols, and controlled dosing inherently limits causal inference. While comprehensive PPS and close medical oversight may enhance participant safety and data quality, they do not eliminate confounding factors related to self-selected PED use, polypharmacy, and heterogeneous exposure histories.

Accordingly, findings derived from this setting should be interpreted as hypothesis-generating rather than confirmatory. The value of TEG lies primarily in structured observation and risk characterization, rather than in validating performance enhancement strategies or defining safety thresholds.

For this reason, eligibility decisions in enhanced athletes cannot currently rely on PED-specific validated cut-offs, but must instead be guided by existing sports cardiology principles, individualized risk stratification, and longitudinal reassessment.

### Cardiovascular Scientific Gaps Beyond TEG’s Reach

Although SCD cannot be excluded, its occurrence during TEG competition is likely to be rare, partly due to the limited number of participants and the inherently low incidence of SCD even in populations with documented PEDs exposure. The currently announced competing categories include 50 m and 100 m freestyle swimming, 50 m and 100 m butterfly swimming, 100 m sprint, 100 m/110m hurdles, snatch, and clean & jerk [1]. From the CV perspective, these subdisciplines are considered power-type sports, requiring relatively short bouts of maximal muscle activation and bearing minor endurance implications. Notably, mixed and endurance, but not power-type sport disciplines, have been associated with more SCD events in competition, introducing additional confounding factors into the interpretation of safety findings [3, 131, 134, 138]. Currently, there is no evidence suggesting that the risk of acute CV events during competition in these athletes differs substantially from that previously described in other athletic populations. Instead, available data indicate that the risk is more strongly influenced by cumulative exposure history rather than a single competitive event [139, 140]. In this context, factors such as polypharmacy, supraphysiologic dosing, duration of (ab)use, and pre-existing CV risk play a central role. Therefore, it is unlikely that athletes with relatively recent or time-limited PED exposure—particularly if explicitly undertaken for a single competition—would already manifest the full spectrum of adverse CV effects associated with long-term use. The preparation for the event will span approximately 3–6 months, a limited timeframe to assess longitudinal health risks and the factors mitigating them. Long-term health risks may be elucidated with ongoing follow-up of the participants. Specifically, major gaps could be filled with longitudinal CMR and CCTA studies focusing on myocardial tissue parameters, myocardial strain measurements, and coronary plaque formation and quality. In parallel, structured longitudinal rhythm monitoring, including ambulatory and exercise-induced electrocardiographic assessment, may be essential to better understand the evolution, burden, and clinical significance of arrhythmic manifestations in pharmacologically enhanced athletes.

Finally, the effects of PEDs are heavily understudied in women. As women’s participation in pharmacologically enhanced sport increases, dedicated sex-specific CV research is of key importance.

## Conclusion

TEG presents plausible and non-negligible CV risks and should be approached with appropriate scientific distance and caution. The unfavorable reputation of PEDs is grounded in well-documented health risks and consistently detrimental findings reported in excessive or prolonged use. At the same time, robust granular data describing longitudinal clinical CV outcomes and prognostically relevant biomarkers in PED-exposed individuals remain limited.

The concept of athletes openly using PEDs has understandably sparked intense debate among stakeholders. Importantly, this review does not seek to endorse such practices. Rather, it acknowledges that the availability and use of so-called “classic” PEDs are increasing in the general population, particularly among recreational and sub-elite athletes, and increasingly also among female athletes, thereby creating a growing clinical reality for CV care. In this context, a dedicated, risk-adapted cardiovascular PPS framework is a critical prerequisite. Enhanced athletes should not be considered physiologically equivalent to natural athletes, and systematic baseline assessment followed by longitudinal monitoring is essential to identify early or subclinical cardiac alterations and to guide clinical decision-making. At present, however, no dedicated evidence-based eligibility thresholds exist for pharmacologically enhanced athletes, and clinical judgement must therefore rely on established sports cardiology criteria, individualized risk stratification, and serial reassessment.

If conducted under rigorous, multidisciplinary medical oversight, TEG may offer a high-risk observational setting capable of generating clinically relevant data that have not previously been available. Such data could inform downstream research aimed at elucidating the reversibility, progression, and long-term prognostic significance of PED-associated CV changes, using advanced imaging and circulating biomarkers.

An additional challenge in this field is the combination of sociocultural and ethical barriers surrounding research on pharmacological performance enhancement in athletes. Participation in such studies may carry negative moral judgment from the sporting community, potentially discouraging structured scientific investigation. As a result, a significant proportion of the available evidence derives from self-selected or high-risk populations using these substances without medical supervision, which may introduce selection bias and limit the generalizability of current findings.

Finally, TEG must not be interpreted as a proof-of-concept for safe enhancement. It should instead be regarded as a setting that demands exceptional ethical scrutiny, transparency, and long-term accountability, with CV research firmly centered on risk characterization, harm recognition, and safety.

## Data Availability

Not applicable.

## Abbreviations

AAS: Androgenic anabolic steroid
CCTA: Coronary computed tomography angiography
CMR: Cardiovascular magnetic resonance
CV: Cardiovascular
ECV: Extracellular volume
ESA: Erythropoiesis-stimulating agent
GIP: Gastric inhibitory peptide
GLP-1: Glucagon-like peptide 1
HF: Heart failure
LVEF: Left ventricular ejection fraction
MI: Myocardial infarction
PED: Performance-enhancing drug
PPS: Pre-participation screening
SCD: Sudden cardiac death
TEG: The Enhanced Games
WADA: World Anti-Doping Agency
